# Development of a Novel Human Hepatoma Cell Line Supporting the Replication of a Recombinant HBV Genome with a Reporter Gene

**DOI:** 10.3390/v18020187

**Published:** 2026-01-30

**Authors:** Shotaro Kawase, Tetsuro Shimakami, Kazuyuki Kuroki, Kazuhisa Murai, Masaya Funaki, Mika Yoshita, Masaki Kakuya, Reo Suzuki, Ying-Yi Li, Dolgormaa Gantumur, Taro Kawane, Koji Matsumori, Kouki Nio, Kazunori Kawaguchi, Hajime Takatori, Masao Honda, Taro Yamashita

**Affiliations:** 1 Department of Gastroenterology, Kanazawa University Graduate School of Medicine, 13-1 Takara-machi, Kanazawa 920-8641, Ishikawa, Japan; skawase.0806@gmail.com (S.K.); kkuroki@staff.kanazawa-u.ac.jp (K.K.); funachan1@gmail.com (M.F.); muharu1125@gmail.com (M.Y.); m.kakuya.39@gmail.com (M.K.); reonandesu@gmail.com (R.S.); liyingyi@staff.kanazawa-u.ac.jp (Y.-Y.L.); dolgormaa6275@gmail.com (D.G.); barutoest57@gmail.com (T.K.); matsumorikoji@gmail.com (K.M.); nio@m-kanazawa.jp (K.N.); kawaguchi@m-kanazawa.jp (K.K.); takatori@m-kanazawa.jp (H.T.); taroy62m@staff.kanazawa-u.ac.jp (T.Y.); 2 Advanced Science Research Center, Kanazawa University, 13-1 Takara-machi, Kanazawa 920-0934, Ishikawa, Japan; k.murai.0612@gmail.com (K.M.); mhonda@m-kanazawa.jp (M.H.)

**Keywords:** hepatitis B virus (HBV), covalently closed circular DNA (cccDNA), HiBiT, NanoLuc^®^ Binary Technology (NanoBiT), HepG2, antiviral screening

## Abstract

Hepatitis B virus (HBV) remains a major global health threat because covalently closed circular DNA (cccDNA) persists in hepatocytes and limits the efficacy of current antiviral therapies. Effective HBV research and drug screening require culture models that recapitulate the complete viral life cycle and allow for quantitative monitoring of replication. In this study, an 11-amino acid luminescent reporter, HiBiT, was inserted at multiple sites within the preS1 region of a genotype D HBV genome, and the C terminus of preS1 was identified as optimal for maintaining robust replication. We then established HepG2-B4 cells stably replicating HiBiT-HBV with HiBiT at the preS1 C terminus. Extracellular HiBiT activity and supernatant levels of HBV-DNA, HBsAg, and HBcAg increased continuously until day 42 and were reduced by nucleos(t)ide analog treatment, and cccDNA was confirmed by Southern blot analysis. Supernatants from HepG2-B4 cells infected naïve HepG2-NTCP cells and primary human hepatocytes, as shown by extracellular HiBiT activity. Transcriptome analysis revealed distinct gene expression changes in HepG2-B4 cells compared with parental HepG2 cells. These findings indicate that the HepG2-B4 system provides a rapid, quantitative, and scalable platform for HBV replication and infection studies and is suitable for mechanistic investigations and high-throughput antiviral screening.

## 1. Introduction

Hepatitis B virus (HBV) infection is a serious global health problem, with an estimated 254 million people chronically infected worldwide in 2022 [1]. Current antiviral therapies, including nucleos(t)ide analogs and interferon (IFN), can delay the onset of HBV-associated liver cirrhosis and hepatocellular carcinoma [2]. However, these agents cannot achieve a cure due to the persistence of covalently closed circular DNA (cccDNA) in infected hepatocytes. The development of antiviral strategies that target and eliminate cccDNA is therefore a critical priority [3]. Progress in this area requires robust HBV culture systems that reproduce the complete viral life cycle and enable the straightforward quantitative monitoring of infection and replication.

Reporter-expressing recombinant HBV provides an attractive solution, as reporter signals can serve as direct indicators of infection and replication, avoiding labor-intensive genome or protein assays. However, the construction of such recombinant HBV poses major challenges. The viral genome contains four overlapping open reading frames and multiple cis-regulatory elements, leaving little tolerance for additional sequences. HBV capsids also impose a strict constraint on genome size, such that even small insertions can impair packaging [4,5]. Moreover, functions disrupted by insertions are difficult to restore, even with protein complementation in trans [6].

To overcome these limitations, we explored the use of NanoLuc^®^ Binary Technology (NanoBiT) [7]. This system is based on a split luciferase composed of two subunits: the high-affinity NanoBiT tag (HiBiT) and the large NanoBiT fragment [8]. Although neither subunit alone is active, their reconstitution restores NanoLuc enzymatic activity in cells or in vitro. HiBiT is an 11-amino acid peptide (VSGWRLFKKIS) that has already been used to tag viral genomes and monitor replication in flaviviruses such as dengue virus, Japanese encephalitis virus, hepatitis C virus, and bovine viral diarrhea virus [9].

In our earlier work [10], we inserted the HiBiT sequence at the N terminus of preS1 within a 1.2-fold genotype C HBV plasmid. The resulting recombinant HiBiT-HBV, generated through the transfection of HepG2-derived cells, produced infectious culture-derived virions (HiBiT-HBVcc) that replicated efficiently in primary human hepatocytes (PHHs) from humanized PXB mice. HiBiT activity was easily detected in both cells and supernatants, reflecting viral replication and release. Moreover, HiBiT-HBVcc amplified in PHHs was able to infect naive PHHs, confirming that infection and replication were maintained. Despite these advantages, the system had critical drawbacks. Viral titers in the supernatant were difficult to quantify accurately, as plasmid DNA remnants interfered with measurements. In addition, HiBiT-HBVcc displayed lower fitness than wild-type HBVcc, showing reduced infectivity and impaired relaxed circular DNA (rcDNA) synthesis. Although the system performance was adequate in PHHs, it was inefficient for monitoring infection in HepG2-NTCP cells, which stably express the HBV entry receptor NTCP.

To address these limitations, we re-engineered HiBiT insertion sites within the preS1 region, established a HepG2 cell line that stably supports HiBiT-HBV replication, and systematically evaluated this new platform.

## 2. Materials and Methods

### 2.1. Plasmid

The 1.1-fold HBV genome was amplified from HepG2.2.15 cells [11], which harbor HBV of genotype D, subtype ayw (GenBank accession number U95551), and inserted into pCMV-Script EX (Agilent Technologies, Santa Clara, CA, USA). This plasmid was designated pCSHBY. In this plasmid, the HBV pregenomic RNA (pgRNA) is transcribed under the control of a CMV promoter. This plasmid contains the neomycin-resistance gene. The HiBiT-coding sequence (5′-GTG AGC GGC TGG CGG CTG TTC AAG AAG ATC AGC-3′) was inserted or substituted into the N or C terminus of preS1 of pCSHBY using the GeneArt Seamless Cloning and Assembly Enzyme Kit (Thermo Fisher Scientific, Waltham, MA, USA). The sequence of the PCR-amplified regions was confirmed using Sanger sequencing.

### 2.2. Cells

The pCI-Neo plasmid encoding HiBiT-HBV with HiBiT at the N terminus of preS1 was transfected into HepG2 cells using Lipofectamine 3000 (Thermo Fisher Scientific, Waltham, MA, USA) according to the manufacturer’s instructions. Neomycin-resistant HepG2 cells were selected in medium containing 500 μg/mL G418 (Thermo Fisher Scientific, Waltham, MA, USA). Several G418-resistant cell lines were expanded clonally. The HepG2-B4 cell line, which showed the highest HiBiT activity, was used for further analysis.

Sodium taurocholate cotransporting polypeptide (NTCP) is a well-known HBV receptor. The HepG2-NTCP-sec cell line [12], which stably overexpresses human NTCP, was used in the present study. The cells were cultured in Dulbecco’s modified Eagle’s medium supplemented with 10% fetal bovine serum, 1% L-glutamine, and 1% penicillin/streptomycin (all from Thermo Fisher Scientific) in a humidified atmosphere of 5% CO_2_ at 37 °C. PXB cells, which are PHHs, were isolated from chimeric PXB mice with humanized livers; the purity of the isolated human hepatocytes exceeded 90% [13]. PXB mice are prepared by the transplantation of human hepatocytes into urokinase-type plasminogen-activator transgenic/SCID mice; the transplanted human hepatocytes maintain their original characteristics in the host mouse liver [14]. The PXB cells were purchased from PhoenixBio (Hiroshima, Japan) and cultured according to the manufacturer’s instructions.

### 2.3. Production of HBVcc

Viral particles in supernatants from HepG2 cells stably replicating HBV were precipitated using a PEG Virus Precipitation Kit (BioVision, Milpitas, CA, USA). HepG2-NTCP-sec cells or PXB cells were inoculated overnight with HBVcc for infection at 50,000 gEq/cell in the presence of 4% PEG 8000 (Merck, Darmstadt, Germany) and 2% DMSO.

### 2.4. HiBiT Assay

Extracellular HiBiT activity was determined using the Nano Glo HiBiT Lytic Detection System (Promega, Madison, WI, USA) with the GloMax-Multi + Detection System (Promega). To determine intracellular HiBiT activity, all cell lysates from each well of a 96-well plate were assessed. For the extracellular assay, 50 µL of medium was analyzed.

### 2.5. Reagents

Myrcludex B (MyrB) was synthesized using COSMO BIO (Tokyo, Japan). Entecavir (ETV), tenofovir disoproxil fumarate (TDF), tenofovir alafenamide fumarate (TAF), and adefovir (ADV) were purchased from Selleck Chemicals (Houston, TX, USA) and dissolved in DMSO. IFNα2b was purchased from Merck.

### 2.6. Southern Blot Analysis

On days 7 and 14 after HepG2-B4 cell seeding, cccDNA was extracted using Hirt’s protein-free DNA extraction procedure [15]. Briefly, the cells were treated with SDS and mixed with a high concentration of NaCl to precipitate high-molecular-weight cellular chromatin and DNA-bound protein. The cccDNA-containing supernatant was collected after centrifugation and purified using a QIAamp DNA Blood Mini Kit (Qiagen, Hilden, Germany). Following treatment with or without heat denaturation, the isolated cccDNA was separated by electrophoresis on a 1.0% agarose gel and transferred to a Hybond-N blotting membrane (Cytiva, Marlborough, MA, USA). Then, pUC19-HBV, which is 3.2 kb in length and includes a 540 bp HBV sequence, was used as a size marker for rcDNA, linearized DNA, and cccDNA because it has a single recognition site for the nicking endonuclease Nb.BbvCI (New England Biolabs, Ipswich, MA, USA) and a single recognition site for ScaI (Takara Bio, Inc., Kusatsu, Japan). Thus, plasmids digested with Nb.BbvCI, ScaI, ScaI with heat denaturation, and no restriction enzyme can serve as size markers for rcDNA, linearized DNA, ssDNA, and rcDNA/cccDNA, respectively. Plasmids treated under these conditions were mixed and then subjected to gel electrophoresis and Southern blotting. The membrane was immobilized using an ultraviolet crosslinker and hybridized with a P32-α-dCTP-labeled full-length HBV-DNA fragment probe (GenBank accession number AB246345) generated with a Random Primer DNA Labeling Kit Ver.2 (Takara Bio). Detection was performed with a BAS Imaging Plate (BAS IP MS 2025 E; Cytiva) and analyzed using a Typhoon FLA7000 scanner (Cytiva).

### 2.7. Quantification of Extracellular HBV-DNA

Extracellular HBV-DNA levels were determined using an HBV qPCR Kit (Kubix, Hakusan, Japan).

### 2.8. Quantification of PreS Antigen and HBcAg

PreS antigen and HBcAg levels were measured using an HBs Pre-S Antigen Quantitative ELISA Kit (Beacle, Kyoto, Japan) and a QuickTiter HBV Core Antigen ELISA Kit (Cell Biolabs, Inc., San Diego, CA, USA), respectively.

### 2.9. Immunostaining

B4 cells were visualized using a rabbit polyclonal anti-HBc antibody (HBP-023-9; Austral Biologicals, San Ramon, CA, USA) or anti-HBs preS1 antibody (Beacle, Kyoto, Japan; BCL-ABP1-01) and an Alexa Fluor 594-conjugated anti-rabbit IgG secondary antibody (Thermo Fisher Scientific). Nuclei were stained with DAPI. Imaging was performed using a BIOREVO fluorescence microscope (Keyence Corporation, Osaka, Japan).

### 2.10. RNA-Seq Analysis

Total RNA was extracted from cultured HepG2 and B4 cells using the RNeasy Mini Kit (Qiagen) according to the manufacturer’s instructions. Following extraction, residual genomic DNA was removed by DNase I treatment.

### 2.11. Library Preparation and Sequencing

RNA-seq libraries were prepared using the TruSeq Stranded mRNA Library Prep Kit with a standard protocol. Sequencing was performed on 101 bp paired ends on an Illumina NovaSeq 6000, resulting in 62–63 million reads per sample. The data have been deposited with links to BioProject accession number PRJDB37986 in the DDBJ BioProject database.

### 2.12. RNA-Seq Processing

Raw FASTQ files were processed in the RaNA-seq cloud environment (https://ranaseq.eu (accessed on 22 January 2026)). Adapters and low-quality bases were removed with fastp, and transcript abundances were estimated with Salmon using quasi-mapping against the GRCh38 human reference transcriptome. Gene-level count tables were exported and imported into R 4.4.3 (Bioconductor 3.20) for downstream analysis.

### 2.13. Heatmap Visualization

Counts for HepG2 (TR_M218_001) and B4 (TR_M218_002) were TMM-normalized with edgeR 4.4.2 and converted to log2CPM (prior.count = 1). The top 50 genes, determined using |log2FC| (B4-HepG2), were displayed with ComplexHeatmap 2.18.0; values were row-mean centered and colored on a symmetric blue–white–red scale to indicate deviation from each gene’s mean log2CPM.

### 2.14. Pre-Ranked GSEA (GO:BP)

Per-gene effect sizes were defined as log2(B4 + 1) − log2(HepG2 + 1); genes with mean counts < 10 were filtered out. Gene symbols were mapped to Entrez IDs using org.Hs.eg.db 3.20.0. The pre-ranked list (names = Entrez ID, weights = log2FC) was analyzed with clusterProfiler 4.14.6 gseGO (GO: Biological Process). Redundant significant terms were reduced with the simplify function using GOSemSim 2.32.0 (IC-based “Rel”, cutoff = 0.5). Terms with a false discovery rate (FDR) q < 0.05 were retained, and summary plots were generated with enrichplot 1.26.6 (dotplot, gseaplot2).

## 3. Results

### 3.1. Establishment of the HepG2-B4 Cell Line

The 1.1-fold HBV genome was amplified by PCR from DNA extracted from HepG2.2.15 cells [11], which harbor HBV (genotype D, subtype ayw, GenBank accession number U95551), and inserted into pCMV-Script EX to generate an expression plasmid carrying the 1.1-fold HBV genome. This construct was designated pCSHBY.

In our previous study [10], the HiBiT-coding sequence, consisting of 33 bases and encoding 11 amino acids, was inserted into the N-terminal portion of preS1 of genotype C HBV, adjacent to the NTCP-binding region. Although the recombinant virus was able to infect and replicate in PXB cells, its infectivity and replicative capacity were reduced compared with wild-type virus lacking HiBiT. We therefore speculated that alternative HiBiT insertion sites, other than the N terminus of preS1, might improve the infectivity and/or replicative capacity of the recombinant virus. Furthermore, as the replicative capacity of genotype D HBV is reported to be superior to that of genotype C [16], we used genotype D HBV in this study.

**Figure 1:**
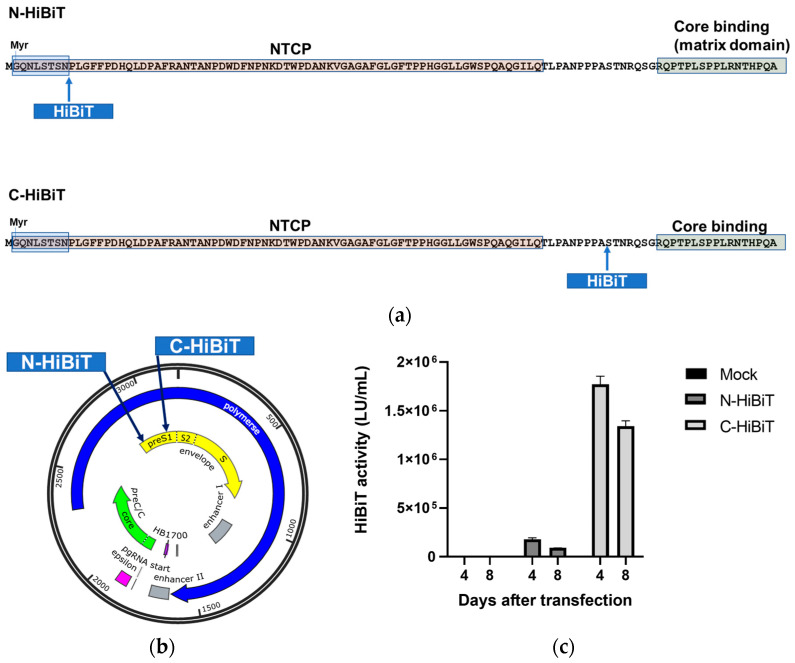
Comparison of HiBiT insertion sites in the preS1 region. (**a**) Schematic illustration of the preS1 region in HBV genotype D plasmid constructs, showing the locations of HiBiT insertion at the N-terminal (N-HiBiT) or C-terminal (C-HiBiT) side of the NTCP-binding region. The preS1 amino acid sequence from HBV genotype D is depicted, with the myristoylation site (Myr), its motif, and the core-binding region highlighted. The light blue and light green boxes indicate the myristoylation and core-binding (matrix) regions, respectively, while the region between these boxes represents the NTCP-binding region. In the N-HiBiT construct (top panel), the HiBiT-coding sequence is inserted immediately after the myristoylation motif at the N terminus. In the C-HiBiT construct (bottom panel), the HiBiT-coding sequence is inserted at the C terminus, adjacent to the core-binding (matrix) domain. (**b**) Schematic representation of the genotype D HBV genome and HiBiT insertion sites. The circular map shows the overlapping open reading frames of polymerase (blue), envelope/preS1–preS2–S (yellow), and precore/core (green), together with key regulatory elements including enhancer I and II and the pregenomic RNA (pgRNA) start site. The positions of the N-terminal (N-HiBiT) and C-terminal (C-HiBiT) HiBiT tag insertions within the preS1 region are indicated by arrows. (**c**) HiBiT activity in supernatants of stable HepG2 cell lines transfected with HBV genotype D plasmids containing HiBiT tags at the N or C terminus of preS1. Culture supernatants were collected at 4 and 8 days after seeding. HiBiT activity (LU/mL) was measured by luminescence assay. Mock control in which HepG2 cells were treated with transfection reagent alone without plasmid DNA, is shown for comparison. Values are shown as mean ± standard deviation of experiments performed using two wells per condition. Note that the mock control values are near zero and overlap with the X-axis. Abbreviations: HBV, hepatitis B virus; NTCP, sodium taurocholate cotransporting polypeptide.

To determine whether insertion at the N- or C-terminal side of the NTCP-binding region was more suitable, the HiBiT sequence was inserted in two ways into the genome of genotype D HBV: (i) at the N terminus of the NTCP-binding region, preserving the critical myristoylation site for HBV entry into hepatocytes [17,18] and ensuring that the overlapping polymerase protein was translated at least at its physiological size, and (ii) at the C terminus of the NTCP-binding region, upstream of the core-binding site matrix domain (Figure 1a). When HepG2 cells were transfected with these plasmids, extracellular HiBiT activity was markedly higher from the construct with C-terminal insertion compared to the N-terminal insertion (Figure 1c). This strongly suggested that the C terminus of the NTCP-binding region was more favorable for HiBiT insertion in genotype D HBV.

**Figure 2:**
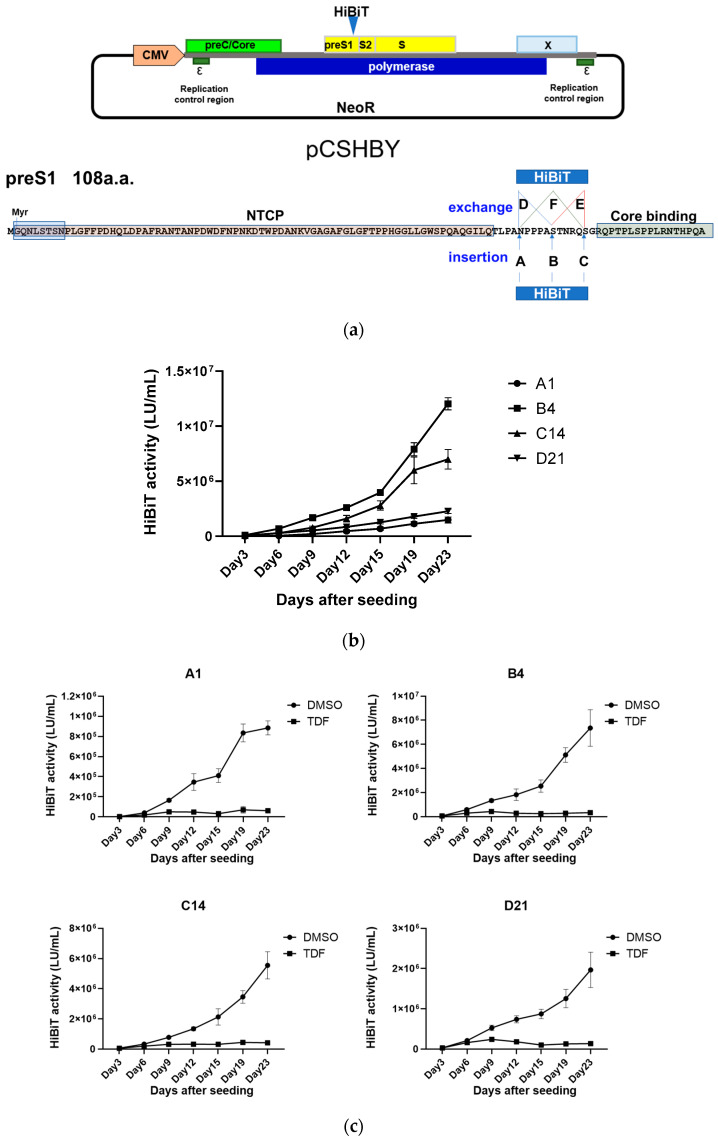
Mapping of HiBiT insertion and substitution/insertion sites in HBV preS1. (**a**) Schematic representation of the pCSHBY plasmid carrying a 1.1-fold genotype D HBV genome. The map shows overlapping open reading frames: polymerase (blue), envelope/preS1–preS2–S (yellow), precore/core (green), and X (light blue). Key regulatory elements include the CMV promoter (orange) and replication control regions (dark green). The HiBiT tag insertion site is indicated by a blue triangle. The preS1 region is marked to show six engineered sites (A–F) where the HiBiT sequence was inserted (A, B, C) or substituted (D, E, F) at the C-terminal region, relative to NTCP-binding and core-binding motifs. Note that stable clones for E and F sites could not be obtained (see text). (**b**) Time course of extracellular HiBiT activity in the culture supernatants of stable HepG2 clones harboring HBV-HiBiT variants (A1, B4, C14, D21). Cells were seeded at equal density and analyzed up to day 23. Values are shown as the mean ± standard deviation of experiments performed using three wells per condition. (**c**) Effect of TDF (50 nM) on HiBiT activity in each clone (A1, B4, C14, D21). Cells were maintained in medium with DMSO or TDF, and extracellular HiBiT activity was measured at the indicated time points. Values are shown as the mean ± standard deviation of experiments performed using three wells per condition. Abbreviations: HBV, hepatitis B virus; NTCP, sodium taurocholate cotransporting polypeptide; TDF, tenofovir disoproxil fumarate.

To further identify the most suitable position for HiBiT insertion or substitution at the C terminus of preS1, the HiBiT coding sequence was introduced at six distinct sites (A–F) within the 1.1-fold genotype D HBV genome in pCSHBY (Figure 2a). Following transfection of each plasmid into HepG2 cells, G418 selection was performed to isolate clones harboring integrated HBV genomes. No G418-resistant clones were obtained from the constructs with insertions at positions E and F. Ultimately, four stable cell lines were established: A1 (insertion at position A), B4 (insertion at position B), C14 (insertion at position C), and D21 (substitution at position D). These cell lines exhibited comparable growth rates.

When equal numbers of cells were seeded and extracellular HiBiT activity was monitored for 23 days, B4 cells displayed the highest HiBiT activity (Figure 2b). In all four cell lines, treatment with the nucleotide analogue TDF greatly reduced extracellular HiBiT activity (Figure 2c), indicating that this signal reflected HBV replication. We selected the B4 cell line, hereafter designated HepG2-B4, for further characterization.

### 3.2. Long-Term Culture of HepG2-B4 Cells and the Effect of Nucleos(t)ide Analogs

**Figure 3:**
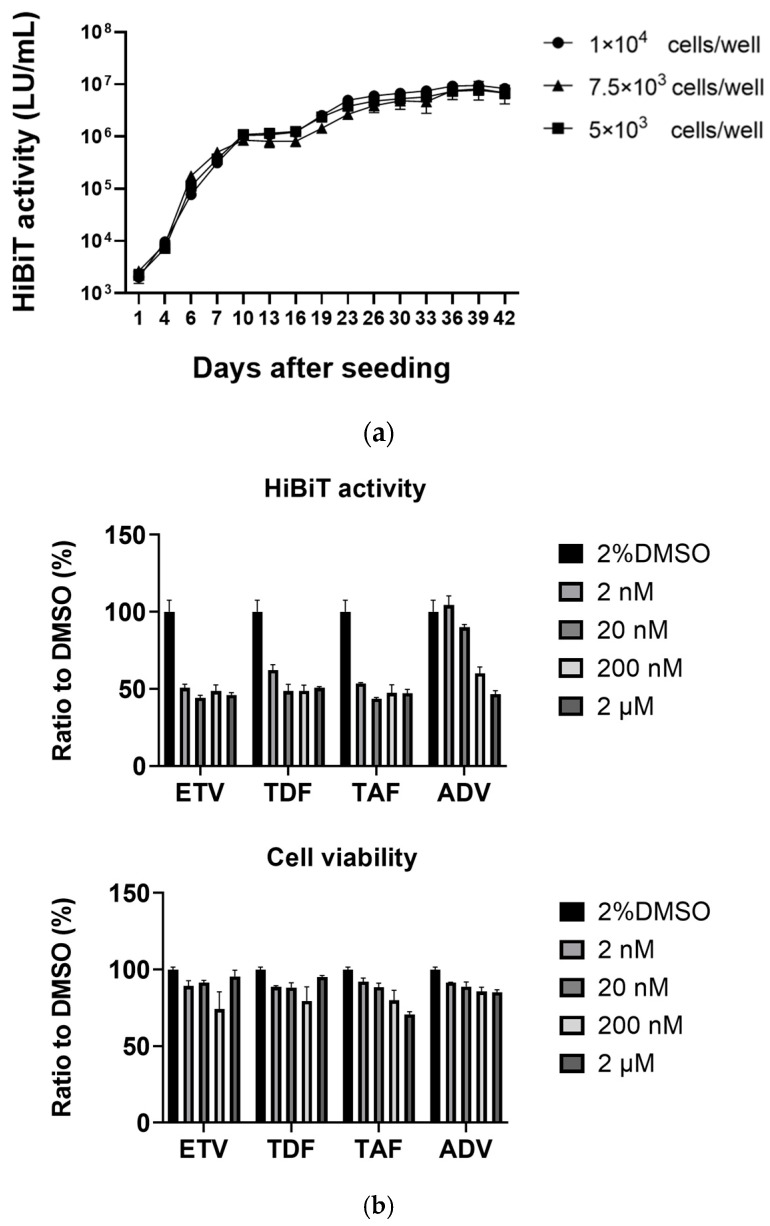
Evaluation of long-term HiBiT activity and sensitivity to nucleos(t)ide analogs in HepG2-B4 cells. (**a**) Time course of extracellular HiBiT activity in HepG2-B4 cells seeded at different densities (1 × 10^4^, 7.5 × 10^3^, or 5 × 10^3^ cells/well) and maintained in 2% DMSO. Culture supernatants were replaced every 2–4 days, and HiBiT activity (LU/mL) was measured up to 42 days. Values are shown as the mean ± standard deviation of experiments performed using two wells per condition. (**b**) The upper graph shows the effect of various concentrations of nucleos(t)ide analogs (ETV, TDF, TAF, ADV) on extracellular HiBiT activity in HepG2-B4 cells. Cells were maintained in medium containing 2% DMSO or the indicated drug concentrations, and HiBiT activity was measured after 25 days. Results are shown as percentages relative to the DMSO (2%) control group (set at 100%). The lower graph shows the cell viability of nucleos(t)ide analogs in HepG2-B4 cells, assessed by the Cell Counting Kit-8 (CCK-8) according to the manufacturer’s instructions. Cells were treated as described in the upper panel. Cell viability was evaluated on day 25. Results are shown as percentages relative to the DMSO (2%) control group (set at 100%) and represent the mean ± standard deviation of experiments performed using three wells per condition. Abbreviations: ADV, adefovir; ETV, entecavir; TAF, tenofovir alafenamide fumarate; TDF, tenofovir disoproxil fumarate.

HepG2-B4 cells were seeded at different densities (5 × 10^3^ cells/well, 7.5 × 10^3^ cells/well, and 1 × 10^4^ cells/well) in 96-well plates with 2% DMSO. Supernatants were collected and replaced with fresh medium every 2–4 days, and extracellular HiBiT activity was measured. HepG2-B4 cells could be maintained in the presence of 2% DMSO without passaging for at least 42 days while showing increasing extracellular HiBiT activity (Figure 3a). The extracellular HiBiT activity rose sharply during the first 10 days after seeding, followed by a more gradual rise thereafter.

Next, we evaluated the effects of several nucleos(t)ide analogs on HiBiT activity and cell viability. HepG2-B4 cells were treated with ETV, TDF, TAF, or ADV at concentrations ranging from 2 nM to 2 μM for 25 days, with regular medium replacement. On day 25, extracellular HiBiT activity and cell viability were assessed. No significant effects on cell viability were observed across the concentrations tested. While ADV showed only weak inhibitory activity, this is consistent with its lower intrinsic in vitro antiviral potency compared with the other nucleos(t)ide analogs reported previously [19,20,21,22]. In contrast, ETV, TDF, and TAF markedly suppressed extracellular HiBiT activity; however, in these three drugs, approximately 50% of the HiBiT signal still remained even at the highest concentrations, representing a basal HiBiT signal that is likely attributable to expression from HBV sequences integrated into the genome of HepG2-derived cells rather than being fully suppressible by nucleos(t)ide analogs [23,24]. Taken together, these findings suggest that HepG2-B4 cells provide a useful system for antiviral screening, in which extracellular HiBiT activity reflects drug-sensitive HBV replication superimposed on a basal level of signal derived from integrated HBV DNA [23,24].

### 3.3. Extracellular HBV Markers

**Figure 4:**
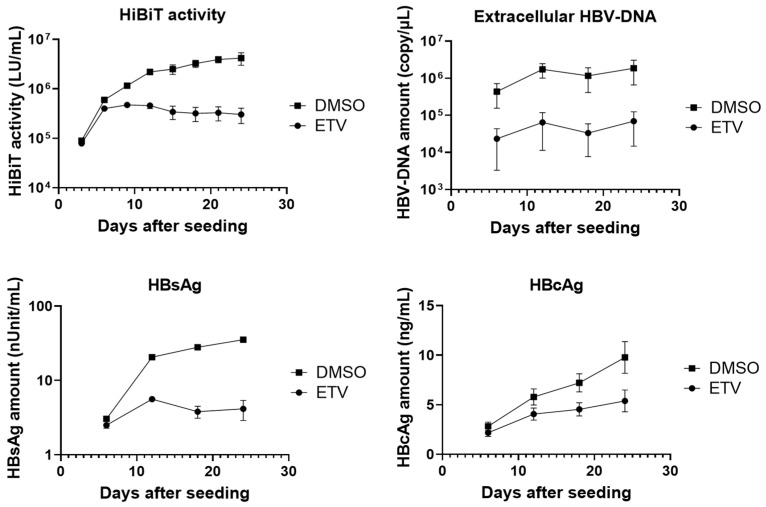
Effects of ETV on extracellular HiBiT activity and HBV markers in culture supernatants. HepG2-B4 cells were treated with DMSO (control) or ETV, and supernatants were collected at indicated time points after seeding. HiBiT activity (LU/mL) was measured by a luminescence assay using three wells per condition. Extracellular HBV-DNA (copies/μL) was quantified using real-time PCR using duplicate measurements from three wells per condition. HBsAg (nUnit/mL) was determined by ELISA using duplicate measurements from two wells per condition, and HBcAg (ng/mL) was determined by ELISA using triplicate measurements from two wells per condition. Values are shown as the mean ± standard deviation. Abbreviations: ELISA, enzyme-linked immunosorbent assay; ETV, entecavir; HBcAg, HBV core-related antigen; HBsAg, hepatitis B surface antigen; HBV, hepatitis B virus.

Next, we quantified several HBV markers in the culture supernatant of HepG2-B4 cells. After seeding, the cells were maintained with or without 200 nM ETV, a nucleoside analog, and extracellular HiBiT activity, HBV-DNA, hepatitis B surface antigen (HBsAg; including large, middle, and small S proteins), and HBV core-related antigen (HBcAg) were measured every 3–6 days until day 24. Extracellular HBV-DNA increased until day 12, slightly declined on day 18, and then increased again. In contrast, extracellular HiBiT activity, HBsAg, and HBcAg continued to increase steadily until day 24. Because HiBiT was expected to be expressed within the large S protein, HiBiT activity correlated closely with HBsAg levels (Figure 4).

ETV treatment markedly suppressed extracellular HiBiT activity, HBV-DNA, and HBsAg. However, its inhibitory effect on HBcAg was limited compared with its suppression of HiBiT activity, HBV-DNA, and HBsAg.

### 3.4. Intracellular HBV-DNA and cccDNA

**Figure 5:**
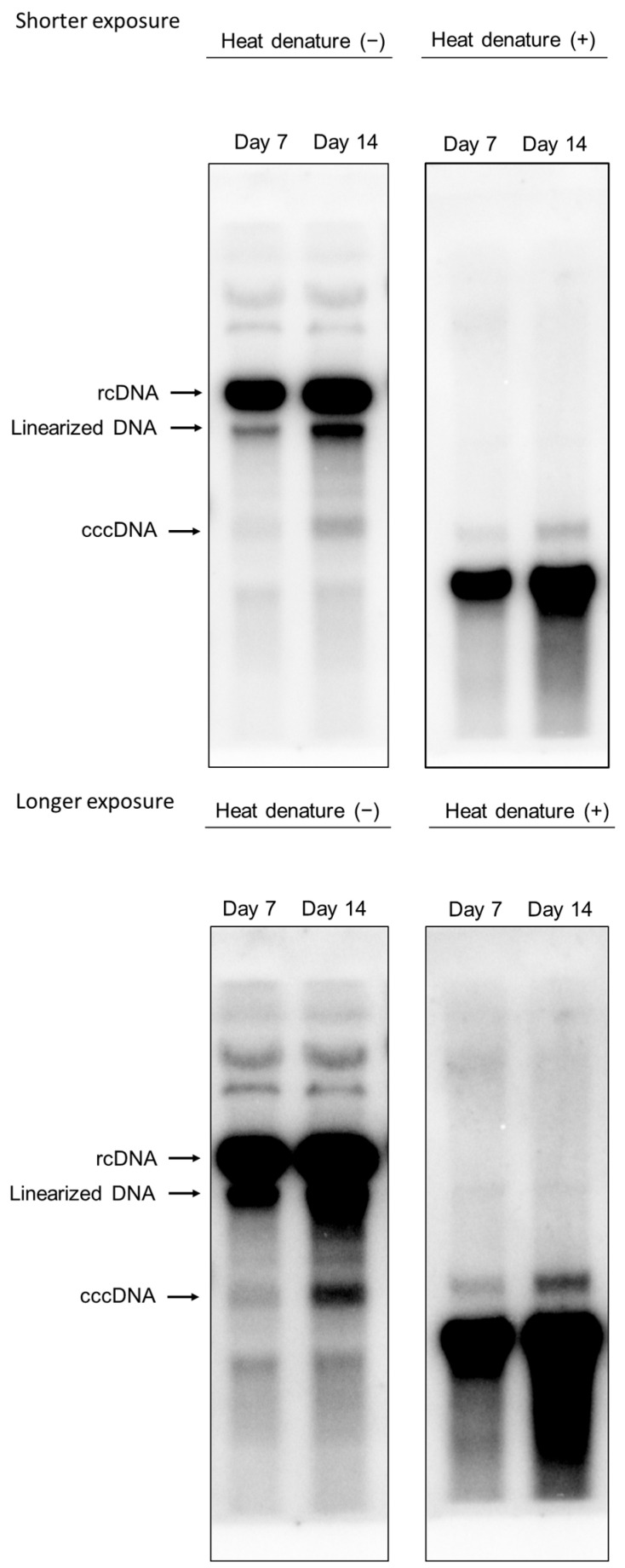
Detection of HBV-DNA species in HepG2-B4 cells by Southern blot analysis following Hirt extraction. HBV-DNA was extracted from HepG2-B4 cells at days 7 and 14 post-seeding using the Hirt method. Samples were analyzed by Southern blotting under conditions with/without heat denaturation, with both shorter (**upper panel**) and longer (**lower panel**) exposure times shown. Signals for cccDNA, rcDNA, and linearized DNA are indicated. The original, uncropped Southern blot images for Figure 5 are provided in the Supplementary Materials as Figure S1. Abbreviations: cccDNA, covalently closed circular DNA; HBV, hepatitis B virus; rcDNA, relaxed circular DNA.

We examined the presence of cccDNA in HepG2-B4 cells. cccDNA was extracted using Hirt’s method, and Southern blot analysis was performed with or without heat denaturation using a full-length HBV probe. This analysis confirmed the presence of cccDNA in cells at days 7 and 14 after seeding, with a higher amount detected at day 14 compared with day 7 (Figure 5).

### 3.5. Immunostaining

**Figure 6:**
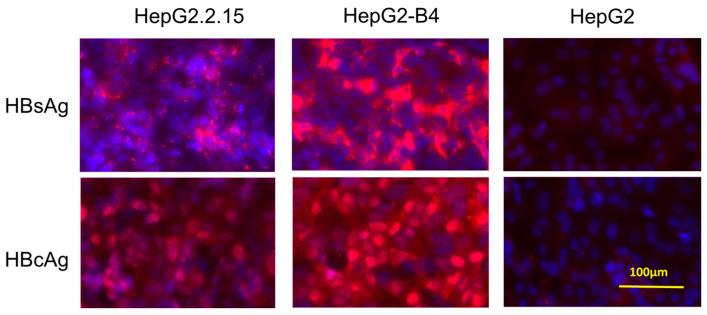
Immunofluorescence detection of HBcAg and HBsAg in HepG2-B4, HepG2.2.15, and parental HepG2 cells. Cells were stained using rabbit polyclonal antibodies against HBcAg and HBsAg, followed by Alexa Fluor 594-conjugated anti-rabbit IgG secondary antibody (red fluorescence). Nuclei were counterstained with DAPI (blue). Abbreviations: HBcAg, HBV core-related antigen; HBsAg, hepatitis B surface antigen.

To confirm the expression of HBsAg and HBV core protein, we performed immunostaining of HepG2-B4 cells 21 days after seeding. The cells were stained with rabbit polyclonal antibodies against HBc and HBs, followed by an Alexa Fluor 594-conjugated anti-rabbit IgG secondary antibody. Nuclei were counterstained with DAPI. As controls, HepG2.2.15 cells and parental HepG2 cells were also examined. Strong expression of HBV proteins was observed in nearly all HepG2-B4 and HepG2.2.15 cells (Figure 6).

### 3.6. Infectivity of HiBiT-HBVcc from HepG2-B4 Cells

**Figure 7:**
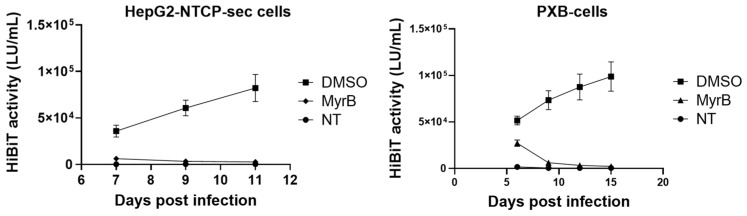
Assessment of HiBiT-HBV infectivity in HepG2-NTCP-sec and PXB cells. The left graph shows the time course of extracellular HiBiT activity in the culture supernatant of HepG2-NTCP-sec cells after infection with HiBiT-HBV (DMSO, MyrB groups) or in non-infected cells (NT group). The right graph shows the same experiment with PXB cells. NT indicates cells that were not infected with HiBiT-HBV. DMSO and MyrB groups were infected with HiBiT-HBV and then treated with DMSO or myrcludex B (MyrB). Values are shown as the mean ± standard deviation of experiments performed using three wells per condition. Abbreviations: HBV, hepatitis B virus; NT, no treatment.

Furthermore, we confirmed the infectivity of HiBiT-HBVcc derived from HepG2-B4 cells. Culture supernatants from HepG2-B4 cells were collected, concentrated using a centrifugal filter, and subsequently used to infect HepG2-NTCP cells and PXB cells. Extracellular HiBiT activity in the supernatant was monitored as an indicator of viral replication. The HiBiT-HBVcc generated from HepG2-B4 cells was able to infect both naïve HepG2-NTCP cells and primary human hepatocytes, as demonstrated by the continuous increase in extracellular HiBiT activity over time. Moreover, the increase in HiBiT activity was abolished when infection was conducted in the presence of the HBV entry inhibitor myrcludex B (Figure 7).

### 3.7. RNA-Seq Analysis of HepG2-B4 Cells

**Figure 8:**
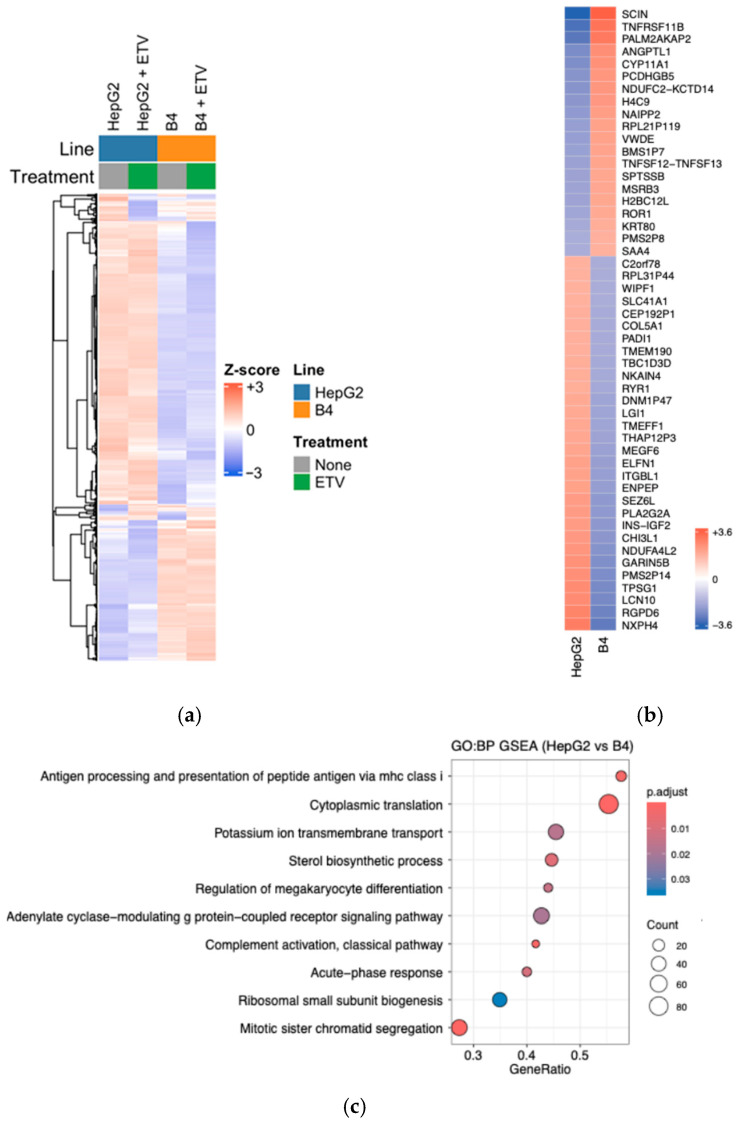

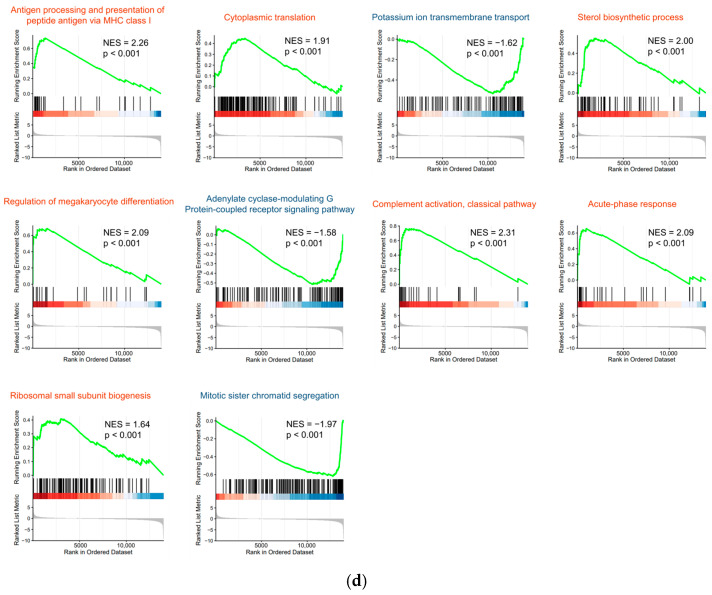
RNA-seq analysis of HepG2-B4 cells. (**a**) The 2000 genes showing the highest expression variance among all samples were selected from the RNA-seq data of HepG2 and HepG2-B4 cells treated or not with ETV. The heatmap shows the hierarchical clustering of genes (rows) with a fixed sample order (columns). Colors represent relative expression levels within each gene (blue = low, white = mean, red = high). Top annotations indicate the cell line (HepG2, blue; B4, orange) and treatment (none, gray; ETV, green). (**b**) Heatmap of the gene expression profiles of HepG2-B4 and HepG2 cells visualizing the top 50 differentially expressed genes between HepG2 and HepG2-B4 cells. The color scale represents the deviation of each gene’s expression from its mean across both cell types, with blue indicating lower expression and red indicating higher expression. (**c**) Dot plot of GSEA. Dot plot showing the Gene Ontology (GO) Biological Process terms that were significantly enriched in HepG2-B4 cells compared to HepG2 cells. The *y*-axis lists the enriched gene sets, while the *x*-axis shows the GeneRatio, which is the proportion of genes from the gene set found in the dataset. The size of each dot corresponds to the number of genes in the gene set; while the legend provides four circles as scale references, actual dot sizes reflect their specific continuous values. The color of the dot represents the adjusted *p*-value (false discovery rate q-value). (**d**) Enrichment plots of GSEA showing pathways upregulated in HepG2-B4 cells (red) and downregulated in HepG2-B4 cells (blue). Enrichment plots detailing the enrichment patterns for the major biological processes shown in Figure 8c. The red-colored biological processes are enriched in HepG2-B4 cells compared to HepG2 cells, whereas the blue-colored biological processes are enriched in HepG2 cells compared to HepG2-B4 cells. The green line in each plot represents the enrichment score, tracking how the gene set is distributed across the ranked list of all genes. The bars at the bottom of each plot indicate the position of the genes within the ranked list, with red bars showing a positive correlation and blue bars showing a negative correlation. The NES and *p*-value displayed on each plot indicate the statistical significance of the enrichment. Abbreviations: ETV, entecavir; GSEA, Gene Set Enrichment Analysis; HBV, hepatitis B virus; NES, Normalized Enrichment Score.

To investigate the impact of HBV replication on gene expression in HepG2 cells, total RNA was extracted from parental HepG2 cells and HepG2-B4 cells and analyzed by RNA-seq. RNA from HepG2 and HepG2-B4 cells treated with ETV was also examined. While the gene expression profiles of parental HepG2 and HepG2-B4 cells were clearly distinct, the suppression of HBV replication using ETV did not revert the expression profile of HepG2-B4 cells to that of parental HepG2 cells (Figure 8a).

A comparison of RNA profiles between parental HepG2 cells and HepG2-B4 cells revealed the significant upregulation in HepG2-B4 cells of biological processes involved in multiple biological processes, including antigen processing and presentation via MHC class I, cytoplasmic translation, sterol biosynthetic processes, regulation of megakaryocyte differentiation, complement activation through the classical pathway, acute-phase response, ribosomal small subunit biogenesis, and mitotic sister chromatid segregation. Conversely, biological processes involved in potassium ion transmembrane transport and adenylate cyclase–modulating G protein–coupled receptor signaling were significantly downregulated in HepG2-B4 cells compared to HepG2 cells (Figure 8b–d).

## 4. Discussion

In this study, we established the HepG2-B4 cell line, which stably replicates HiBiT-tagged HBV with insertion at the C-terminal region of preS1. Previous reports on HiBiT-tagged HBV have examined insertion at the N terminus of either preS1 [10,25] or preS2 [26]; however, all prior studies exclusively focused on genotype C HBV. To our knowledge, our study is the first to demonstrate successful HiBiT insertion at the C terminus of preS1 in genotype D HBV, thereby expanding the applicability of reporter HBV systems. This novel approach provides a new tool for investigating HBV biology and antiviral screening in a previously untested genotype background.

The HBV genome is exceptionally compact, with overlapping open reading frames and essential cis-acting elements, making suitable insertion sites for foreign sequences extremely limited. As observed in previous genotype C studies, we confirmed that both the N- and C-terminal regions of preS1 are permissive for HiBiT-tagging, with the C terminus supporting more efficient replication in genotype D. Importantly, our genotype D construct demonstrated higher viral replication and infectivity in both HepG2-NTCP-sec and PXB cells, whereas prior genotype C constructs frequently exhibited reduced fitness and restricted infection capacity in similar models.

The HepG2-B4 system offers several advantages over conventional HBV models. Our approach eliminates repeated plasmid preparations and reduces the risk of plasmid contamination in viral DNA quantification, streamlining recombinant virus production. In comparison to established cell lines such as HepG2.2.15 [11] and HepAD38 [27], which require time-consuming endpoint assays, the extracellular HiBiT assay in HepG2-B4 enables rapid, non-destructive monitoring and is ideal for high-throughput antiviral drug screening. The marked suppression of HiBiT activity with nucleot(s)ide analogs confirms the suitability of the system for evaluating replication-targeting drugs.

Transcriptome analysis revealed persistent gene expression alterations in HepG2-B4 cells compared to parental HepG2 cells. These alterations were only partially reversed by antiviral treatment with ETV, potentially due to residual HBV replication. This finding parallels clinical observations that the risk of hepatocellular carcinoma persists even in patients undergoing long-lasting nucleot(s)ide analogs therapy.

Despite these strengths, important limitations of the present study should be noted. First, the range of insertion sites compatible with robust virus replication and stable cell line establishment remains very restricted due to the compactness of the HBV genome and the presence of essential regulatory elements. Consistent with previous HBV reporter and vector studies [28,29,30], our findings suggest that only a limited subset of sites can accommodate the HiBiT tag without markedly impairing replication. Therefore, further work is needed to identify and validate additional permissive genomic sites or alternative tagging strategies. Second, while extracellular HiBiT allows for the sensitive monitoring of infection and replication, its readout may not fully recapitulate all steps of the viral lifecycle, particularly those downstream of the inserted region or involving protein–protein interactions altered by the tag. Third, the present study primarily relied on immortalized cell lines. Validation in primary hepatocytes or animal models is warranted before full translational application.

In summary, our study provides the first evidence for successful HiBiT insertion at the preS1 C terminus of genotype D HBV, thereby establishing a robust platform for high-throughput screening and infection assays. Together with the possibility of extending this strategy to additional hepatoma cell lines and other HBV genotypes, these advances will facilitate deeper investigation of HBV pathobiology and may help accelerate the development of new antiviral strategies.

## Data Availability

The data supporting the findings of this study are available within the article and the Supplementary Materials.

## Supplementary Materials

The following supporting information can be downloaded at: https://www.mdpi.com/article/10.3390/v18020187/s1, Figure S1: Original Southern blot images for Figure 5.

## Abbreviations

The following abbreviations are used in this manuscript:

HBV	hepatitis B virus
HBsAg	hepatitis B surface antigen
HBcAg	hepatitis B core antigen
cccDNA	covalently closed circular DNA
NTCP	sodium taurocholate cotransporting polypeptide
