# Supplementary figures and images for: Development of a Novel Human Hepatoma Cell Line Supporting the Replication of a Recombinant HBV Genome with a Reporter Gene
Source: Viruses. 2026 Jan 30;18(2):187. doi: 10.3390/v18020187 (PMC12944927; doi:10.3390/v18020187)

## Supplementary Material

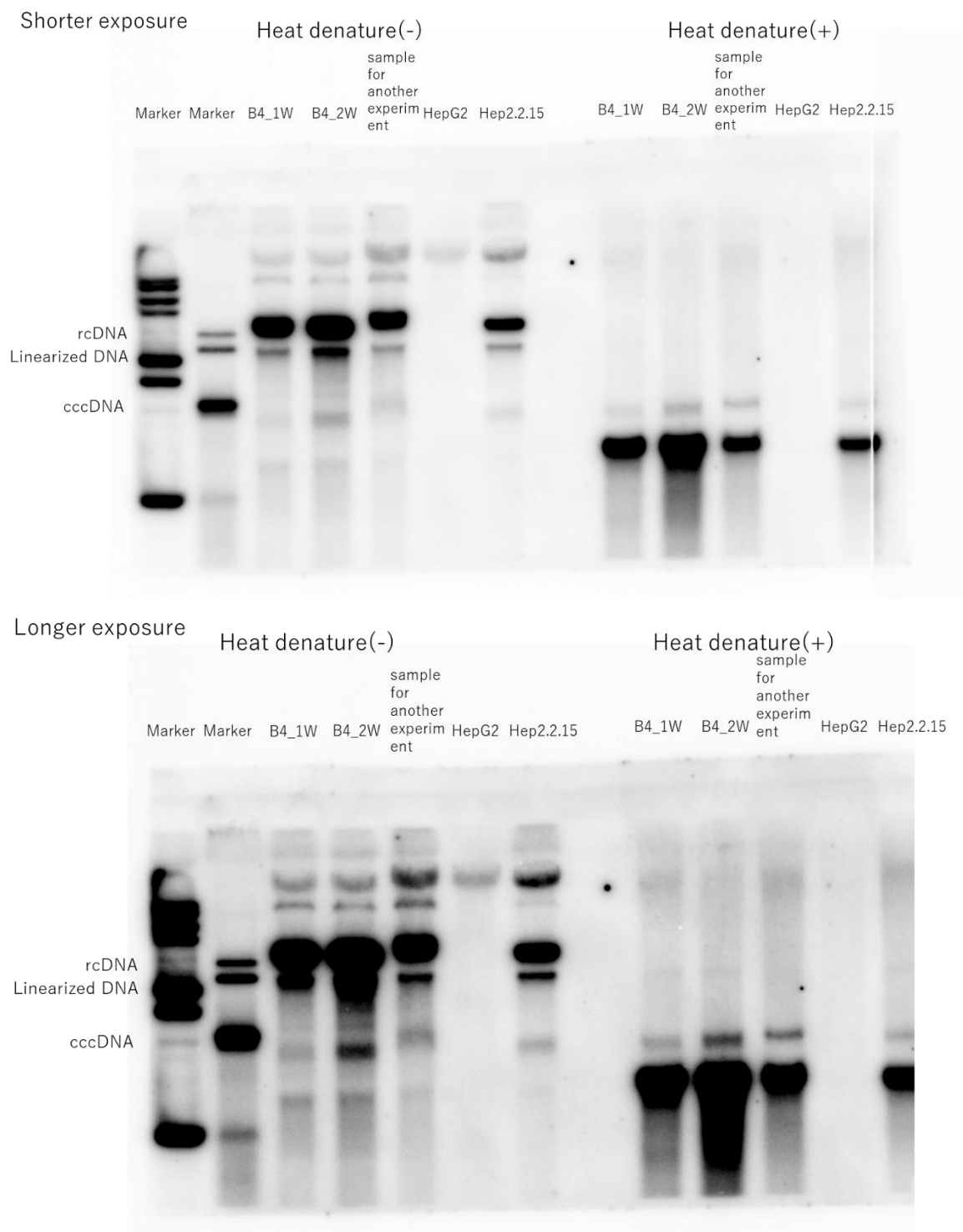

**Figure S1.** Original and uncropped image of Figure 5.

Supplement: Supplementary file 1 [file viruses-18-00187-s001.zip › viruses-4078372-supplementary.pdf]
